# Baseline clinical characteristics and disease burden in patients with paroxysmal nocturnal hemoglobinuria (PNH): updated analysis from the International PNH Registry

**DOI:** 10.1007/s00277-020-04052-z

**Published:** 2020-05-10

**Authors:** Hubert Schrezenmeier, Alexander Röth, David J. Araten, Yuzuru Kanakura, Loree Larratt, Jamile M. Shammo, Amanda Wilson, Gilda Shayan, Jaroslaw P. Maciejewski

**Affiliations:** 1 Institute of Transfusion Medicine, University of Ulm, Ulm, Germany; 2 Institute for Clinical Transfusion Medicine and Immunogenetics, German Red Cross Blood Transfusion Service Baden-Württemberg-Hessen and University Hospital Ulm, Helmholtzstraße 10, 89081 Ulm, Germany; 3 Department of Hematology, West German Cancer Center, University Hospital Essen, University of Duisburg-Essen, Hufelandstr.55, D-45122 Essen, Germany; 4 Department of Medicine, NYU Langone Medical Center, 550 First Avenue, 15-1539, New York, NY 10016 USA; 5 Department of Hematology and Oncology, Osaka University Graduate School of Medicine, C9, 2-2, Yamada-oka, Suita, Osaka, 565-0871 Japan; 6 Department of Medicine, University of Alberta, Edmonton, Alberta Canada; 7 Division of Hematology/Oncology, Rush University Medical Center, 1653 W. Congress Parkway, Chicago, IL 60612 USA; 8 Alexion Pharmaceuticals, Inc., 121 Seaport Boulevard, Boston, MA 02210 USA; 9 Present Address: Sanofi, 50 Binney Street, Boston, MA 02142 USA; 10 Present Address: Alnylam Pharmaceuticals, Inc., 675 West Kendall Street, Cambridge, MA 02142 USA; 11 Department of Translational Hematology and Oncology Research, Taussig Cancer Institute, Cleveland Clinic, 10201 Carnegie Avenue, Cleveland, OH 44195 USA

**Keywords:** Paroxysmal nocturnal hemoglobinuria, Registries, Health-related quality of life, Eculizumab, Thrombosis, Bone marrow failure

## Abstract

The International Paroxysmal Nocturnal Hemoglobinuria (PNH) Registry (NCT01374360) was initiated to optimize patient management by collecting data regarding disease burden, progression, and clinical outcomes. Herein, we report updated baseline demographics, clinical characteristics, disease burden data, and observed trends regarding clone size in the largest cohort of Registry patients. Patients with available data as of July 2017 were stratified by glycosylphosphatidylinositol (GPI)-deficient granulocyte clone size (< 10%, ≥ 10%–< 50%, and ≥ 50%). All patients were untreated with eculizumab at baseline, defined as date of eculizumab initiation or date of Registry enrollment (if never treated with eculizumab). Outcomes assessed in the current analysis included proportions of patients with high disease activity (HDA), history of major adverse vascular events (MAVEs; including thrombotic events [TEs]), bone marrow failure (BMF), red blood cell (RBC) transfusions, and PNH-related symptoms. A total of 4439 patients were included, of whom 2701 (60.8%) had available GPI-deficient granulocyte clone size data. Among these, median clone size was 31.8% (1002 had < 10%; 526 had ≥ 10%–< 50%; 1173 had ≥ 50%). There were high proportions of patients with HDA (51.6%), history of MAVEs (18.8%), BMF (62.6%), RBC transfusion (61.3%), and impaired renal function (42.8%). All measures except RBC transfusion history significantly correlated with GPI-deficient granulocyte clone size. A large proportion of patients with GPI-deficient granulocyte clone size < 10% had hemolysis (9.7%), MAVEs (10.2%), HDA (9.1%), and/or PNH-related symptoms. Although larger GPI-deficient granulocyte clone sizes were associated with higher disease burden, a substantial proportion of patients with smaller clone sizes had history of MAVEs/TEs.

## Introduction

Paroxysmal nocturnal hemoglobinuria (PNH) is a rare, acquired, potentially life-threatening hematologic disorder characterized by chronic, intravascular hemolysis caused by uncontrolled activation of the terminal complement pathway [1–3]. PNH is a clinically heterogeneous disease; in addition to the primary clinical manifestation of chronic intravascular hemolysis, it is associated with bone marrow failure (BMF; e.g., aplastic anemia, myelodysplastic syndrome) and thrombophilia [4–6]. Other disease manifestations include fatigue, abdominal pain, esophageal spasms, male erectile dysfunction, pulmonary hypertension, and renal impairment [1, 2, 6–9].

Thrombotic events (TEs) account for up to 67% of deaths with a known cause in patients with PNH [1, 4, 8, 10, 11]. In a retrospective study, approximately 20% of patients with PNH treated only with supportive care died within 6 years of diagnosis [12].

PNH occurs as a consequence of a somatic mutation in the phosphatidylinositol glycan class A gene in bone marrow stem cells, followed by clonal expansion of the mutated cells. This genetic mutation causes impaired glycosylphosphatidylinositol (GPI) biosynthesis and deficient GPI-anchored complement regulatory proteins on the surface of mature blood cells [4, 13]. GPI-deficient granulocyte clone size, a measure of the relative proportion of GPI-deficient cells, varies widely among patients with PNH [5] and may influence clinical characteristics and disease burden [4–6]. Although patients with larger GPI-deficient granulocyte clone size generally experience more debilitating symptoms and consequences of chronic complement-mediated intravascular hemolysis, limited data suggest that smaller GPI-deficient granulocyte clones may also confer significant disease burden [5, 6]. While larger GPI-deficient granulocyte clone size is associated with increased TE risk, all patients with PNH are at risk for a TE regardless of clone size [6, 11, 14, 15].

The International PNH Registry (NCT01374360) is the largest worldwide observational study of patients with PNH [6], designed to collect disease and treatment-related data that could be used to help optimize management of PNH [16, 17]. Previous analyses of Registry data have demonstrated evidence of the clinical benefit of eculizumab (Soliris®, Alexion Pharmaceuticals, Inc., Boston, MA, USA) for patients who have hemolysis and clinical symptoms indicative of high disease activity (HDA) [16]. Baseline demographic and clinical characteristics of the first 1610 patients enrolled in the Registry have also been previously reported [6].

The objectives of the present analysis are to provide an update on the baseline demographics, clinical characteristics, disease-associated morbidities, PNH symptoms, and health-related QoL in patients, based on a substantially larger cohort of Registry patients, and to assess potential trends in PNH disease burden related to GPI-deficient clone size [6].

## Methods

### Study design and patients

Details regarding the design and patient population included in the International PNH Registry have been previously reported [6]. The Registry is an ongoing, prospective, multicenter, global, observational study that includes patients of any age with a clinical diagnosis of PNH and/or a detectable PNH clone (defined as a population of GPI-deficient granulocytes and/or erythrocytes) of ≥ 0.01% [6]. The institutional review boards (or equivalent) of participating centers approved the Registry, and written informed consent was provided by all patients before study entry [6]. The Registry is sponsored by Alexion Pharmaceuticals, Inc., and is overseen by an Executive Committee of independent international clinical experts in PNH [6].

The current analysis includes patients enrolled in the Registry who had data available as of July 17, 2017. All patients were untreated with eculizumab at baseline, defined as the date of eculizumab initiation for patients who had ever been treated with eculizumab and the date of Registry enrollment for patients who had never received eculizumab treatment.

### Outcomes

Clinical outcomes of interest assessed at baseline included proportions of patients with HDA, history of major adverse vascular events (MAVEs; including TEs) and BMF (including aplastic anemia), red blood cell (RBC) transfusions, concomitant medication use, and presence of physician-reported PNH-related symptoms. TEs include thrombophlebitis/deep vein thrombosis, renal vein thrombosis, renal arterial thrombosis, mesenteric/visceral vein thrombosis, mesenteric/visceral arterial thrombosis, hepatic/portal vein thrombosis, dermal thrombosis, acute peripheral vascular disease occlusion, cerebral arterial occlusion/cerebrovascular accident, cerebral venous occlusion, and pulmonary embolus. MAVEs include TEs as defined above, amputation (nontraumatic, nondiabetic), myocardial infarction, transient ischemic attack, unstable angina, gangrene (nontraumatic, nondiabetic), and other MAVE. Characteristics such as aplastic anemia, hypoplastic anemia, myelodysplastic syndrome, and other bone marrow pathologies were used as a basis to identify history of or concurrent bone marrow failure in Registry patients. Laboratory values were assessed at baseline, defined as the most recent value within 6 months prior to and including the baseline date. HDA at baseline was defined as evidence of hemolysis (elevated lactate dehydrogenase [LDH] ≥ 1.5 times upper limit of normal [ULN]) within 6 months prior to baseline and a history of at least 1 of the following signs or symptoms prior to baseline: fatigue, hemoglobinuria, abdominal pain, dyspnea, anemia (hemoglobin < 100 g/L), MAVEs (including TEs), dysphagia, or erectile dysfunction.

Patient QoL was assessed using the European Organisation for Research and Treatment of Cancer (EORTC) Global Health/QoL subscale, which is rated on a scale ranging from 0 to 100, with higher scores indicating better functioning [18]. Patient-reported fatigue was assessed using the Functional Assessment of Chronic Illness Therapy (FACIT)-Fatigue questionnaire, which is scored on a scale ranging from 0 to 52, with higher scores indicating less fatigue [19, 20]. For QoL measures, baseline was defined as the most recent value within 6 months prior to and including the baseline date.

### Data analysis

Categorical variables were described using frequencies and percentages, and continuous variables were described using mean, standard deviation (SD), median, and interquartile range. For each outcome of interest, analyses were conducted only on the patients who had available data.

Patients whose percent GPI-deficient granulocyte clone size values were available prior to baseline were stratified by clone size category (< 10%, ≥ 10% to < 50%, and ≥ 50%). Potential trends related to clone size were assessed using the Cochran-Armitage trend test of proportion of patients with specified event history by percent of GPI-deficient granulocyte group.

## Results

**Fig. 1:**
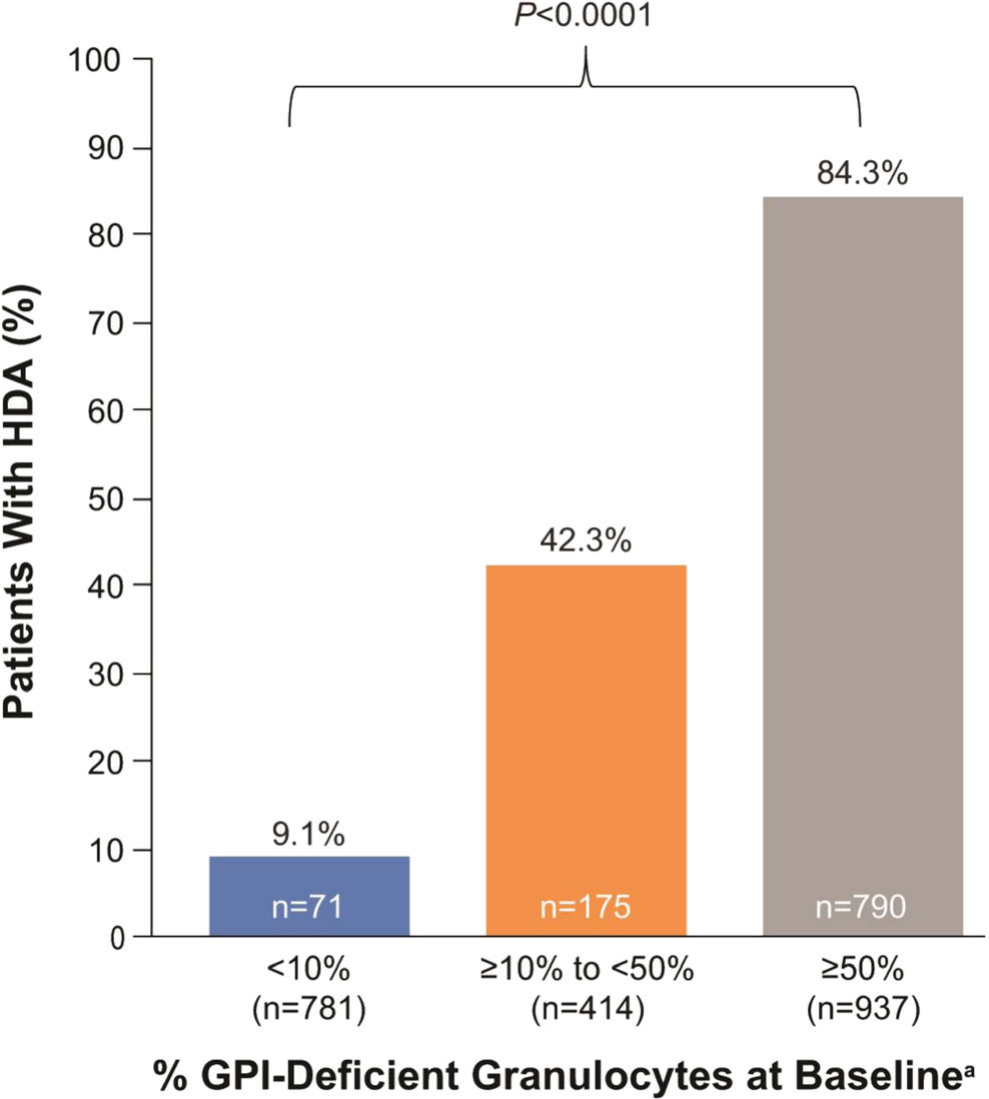
Proportion of patients with HDA stratified by percentage of GPI-deficient granulocytes at baseline ^a^Patients with available data. *GPI* glycosylphosphatidylinositol; *HDA* high disease activity

As of July 17, 2017, a total of 4948 patients were enrolled in the Registry. Of these, 4439 patients had nonmissing data on demographics, enrollment date, and eculizumab status and were included in the study population. Patient demographics are summarized in Table 1. Among the 2701 patients (60.8%) with clone size reported prior to baseline, median (quartile [Q]1, Q3) percent GPI-deficient granulocyte clone size was 31.8% (2.4%, 85.0%). Of those, 1002 patients (37.1%) had GPI-deficient granulocyte clone size < 10%, 526 (19.5%) had clone size ≥ 10% to < 50%, and 1173 (43.4%) had clone size ≥ 50%. More than half of the patients (51.6%) had HDA at baseline, and the proportion of patients with HDA at baseline correlated significantly with GPI-deficient granulocyte clone size category; higher proportions of patients with HDA were observed in subgroups with higher clone size (*P* < 0.0001). However, high proportions of patients with small (< 10%) or mid-size (≥ 10% to < 50%) GPI-deficient granulocyte clones had HDA (9% and 42%, respectively) (Fig. 1).

**Table 1:** Patient demographic characteristics

Characteristic	Overall study population (*n* = 4439)	Patients with GPI-deficient granulocytes data available at baseline (*n* = 2701)
Sex, *n* (%)	*n* = 4439	*n* = 2701
Female	2353 (53.0)	1407 (52.1)
Male	2086 (47.0)	1294 (47.9)
Region, *n* (%)	*n* = 4439	-
Europe	3012 (67.9)	-
North America	640 (14.4)	-
Rest of world	787 (17.7)	-
Race, *n* (%)	*n* = 4420	*n* = 2692
White	3464 (78.4)	2238 (83.1)
Asian	722 (16.3)	320 (11.9)
Black	132 (3.0)	83 (3.1)
Other^a^	102 (2.3)	51 (1.9)
Age at baseline, years	*n* = 4439	*n* = 2701
Mean (SD)	45.1 (18.1)	44.4 (18.6)
Median (Q1, Q3)	43.7 (30.2, 59.6)	42.7 (28.6, 59.8)
Age at disease onset, years	*n* = 4336	*n* = 2667
Mean (SD)	39.3 (18.6)	40.0 (19.0)
Median (Q1, Q3)	35.5 (23.9, 53.4)	36.5 (23.8, 55.2)

*Q* quartile; *SD* standard deviation

^a^Native/aboriginal, Pacific Islander, multiple races, or unlisted

**Fig. 2:**
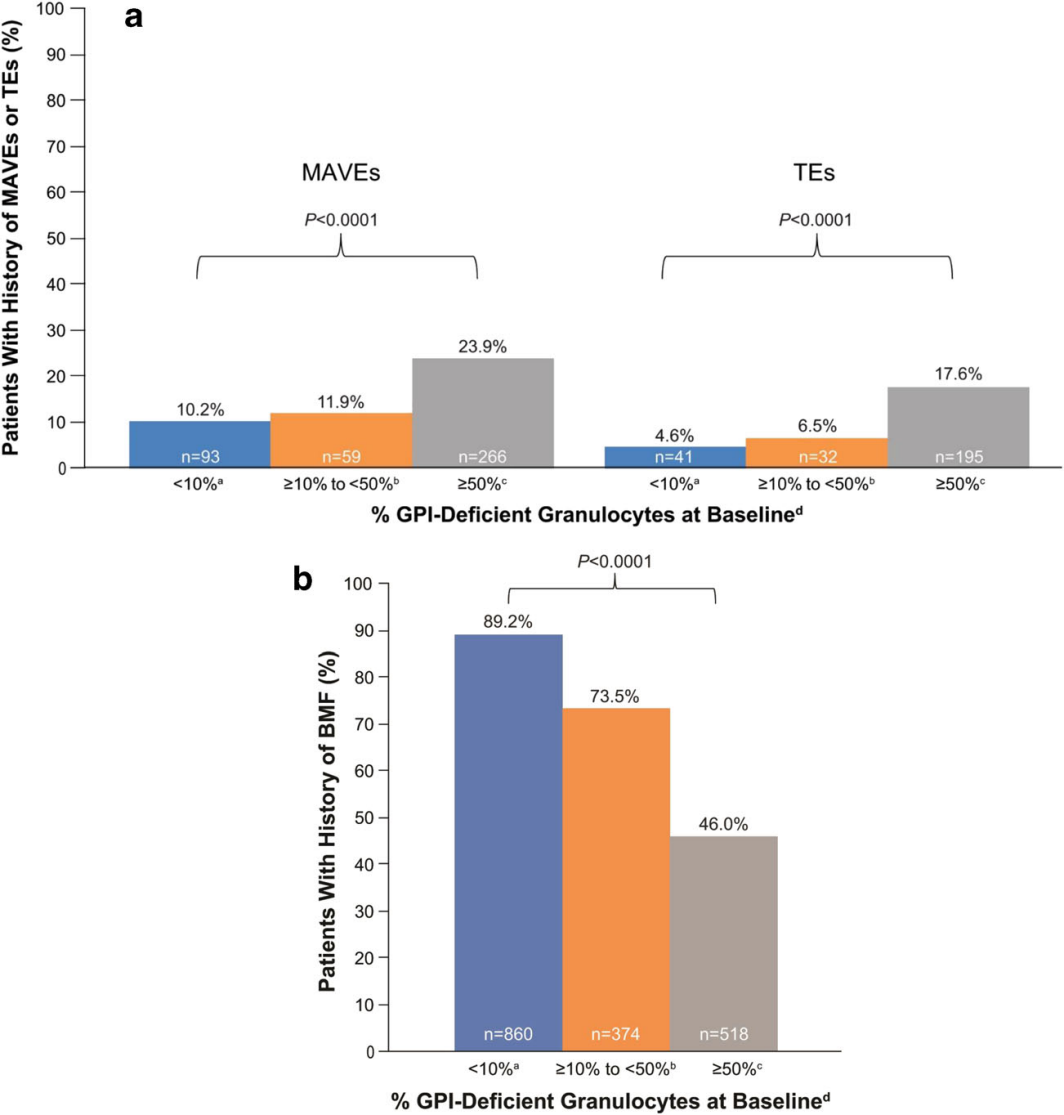
Proportion of patients with a history of (a) MAVEs, TEs (a subset of all MAVEs), or (b) BMF stratified by percentage of GPI-deficient granulocytes at baseline ^a^MAVEs, *n* = 915; TEs, *n* = 899; BMF, *n* = 964. ^b^MAVEs, *n* = 494; TEs, *n* = 495; BMF = 509. ^c^MAVEs, *n* = 1114; TEs, *n* = 1106; BMF, *n* = 1125; ^d^Patients with available data. *BMF* bone marrow failure; *GPI* glycosylphosphatidylinositol; *MAVEs* major adverse vascular events; *TEs* thrombotic events

Overall, 779 of 4134 patients (18.8%) with available data at baseline had a history of MAVEs, including 544 (13.3% of the study population) who specifically experienced TEs. The proportions of patients with a history of MAVEs or TEs at baseline correlated significantly with clone size (Fig. 2a; *P* < 0.0001 for both MAVE and TE). While the proportions of patients with a history of MAVEs and TEs were substantially higher in the cohort with clone size ≥ 50% versus the cohort with clone size ≥ 10% to < 50%, they were only modestly higher in the cohort with clone size ≥ 10% to < 50% versus the < 10% cohort.

Almost 63% of patients had a history of BMF and about 53% (2206/4201) had a history of aplastic or hypoplastic anemia at baseline. The proportions of patients with BMF in each of the cohorts showed an inverse correlation with clone size (Fig. 2b).

Overall, patients had a high burden of disease at baseline, as reflected in the proportions of patients with hemolysis (55.8%), defined by an LDH ratio ≥ 1.5 times ULN at baseline, and impaired renal function (42.8% with estimated glomerular filtration rate [eGFR] < 90 mL/min/1.73 m^2^ and 15.0% with eGFR < 60 mL/min/1.73 m^2^). Median values for all laboratory parameters assessed correlated significantly with GPI-deficient granulocyte clone size category (Table 2). The proportion of patients with impaired renal function (eGFR <90 mL/min/1.73 m^2^) was highest in the cohort with clone size < 10% (51.4%), compared with those with GPI-deficient granulocyte clone sizes of ≥ 10% to < 50% (40.5%) and ≥ 50% (35.0%). Conversely, median laboratory values at baseline for LDH ratio, absolute reticulocyte count, and platelet count showed a significant positive correlation with increasing GPI-deficient granulocyte clone size category (Table 2). There was a significant trend in median hemoglobin levels across GPI-deficient granulocyte clone size cohorts, with the lowest median hemoglobin level observed in the cohort with clone size ≥ 50%.

**Table 2:** Laboratory values and concomitant medication use at baseline

Parameter	All Patients	Patients by % GPI-deficient granulocytes at baseline^a^			
	*n* = 4439	< 10% *n* = 1002	≥ 10% to < 50% *n* = 526	≥ 50% *n* = 1173	*P* value
LDH ratio (LDH/ULN)	*n* = 3085	*n* = 781	*n* = 414	*n* = 937	
Median (Q1, Q3)	1.9 (1.0, 5.1)	0.9 (0.7, 1.1)	1.4 (1.0, 2.3)	4.6 (2.6, 7.3)	< 0.0001
LDH ≥ 1.5 x ULN, *n* (%)	1720 (55.8)	76 (9.7)	200 (48.3)	834 (89.0)	< 0.0001
Hemoglobin (g/L)	*n* = 3581	*n* = 914	*n* = 458	*n* = 1020	< 0.0001
Median (Q1, Q3)	98.0 (82.2, 117.0)	99.4 (80.0, 119.0)	101.0 (87.0, 124.0)	95.0 (81.0, 108.0)	
Absolute reticulocyte count (× 10^9^/L)	*n* = 1954	*n* = 444	*n* = 292	*n* = 664	< 0.0001
Median (Q1, Q3)	87.0 (55.0, 140.0)	56.0 (38.0, 75.0)	75.0 (51.5, 105.5)	124.0 (82.0, 183.0)	
Platelet count (× 10^9^/L)	*n* = 3563	*n* = 884	*n* = 449	*n* = 1026	< 0.0001
Median (Q1, Q3)	110.0 (50.0, 175.0)	59.5 (23.0, 135.0)	81.0 (37.0, 146.0)	129.0 (70.0, 191.0)	
eGFR (mL/min/1.73 m^2^), *n* (%)	*n* = 3257	*n* = 849	*n* = 430	*n* = 934	< 0.0001
< 30	79 (2.4)	12 (1.4)	5 (1.2)	15 (1.6)	
30– < 60	410 (12.6)	122 (14.4)	47 (10.9)	97 (10.4)	
60– < 90	904 (27.8)	302 (35.6)	122 (28.4)	215 (23.0)	
≥ 90	1864 (57.2)	413 (48.6)	256 (59.5)	607 (65.0)	
History of RBC transfusions, *n* (%)	*n* = 3620	*n* = 979	*n* = 476	*n* = 966	0.1204
	2219 (61.3)	589 (60.2)	259 (54.4)	615 (63.7)	
History of anticoagulation therapy^b^, *n* (%)	*n* = 4206	*n* = 952	*n* = 499	*n* = 1127	< 0.0001
	849 (20.2)	49 (5.1)	58 (11.6)	375 (33.3)	
History of immunosuppressive therapy^c^, *n* (%)	*n* = 4232	*n* = 971	*n* = 500	*n* = 1137	< 0.0001
	1642 (38.8)	573 (59.0)	212 (42.4)	302 (26.6)	

*eGFR* estimated glomerular filtration rate; *GPI* glycosylphosphatidylinositol; *LDH* lactate dehydrogenase; *Q* quartile; *RBC* red blood cell; *ULN* upper limit of normal

^a^Patients with available data. ^b^Aspirin, warfarin derivatives, heparin derivatives, or other anticoagulation. ^c^Corticosteroids, cyclosporine, or antithymocyte globulin

In general, 61.3% (2219/3620) of patients had a history of RBC transfusions, 20.2% (849/4206) had a history of anticoagulation therapy, and 38.8% (1642/4232) had a history of immunosuppressive therapy at baseline (Table 2). History of anticoagulant use and history of immunosuppressive therapy were significantly correlated with clone size (*P* < 0.0001), with the proportions of patients with history of anticoagulant use at baseline showing positive correlation with increasing clone size and proportions of patients with a history of immunosuppressive therapy showing inverse correlation with GPI-deficient granulocyte clone size category (Table 2). There was no statistically significant trend in the proportion of patients with a history of RBC transfusions across the three cohorts.

**Fig. 3:**
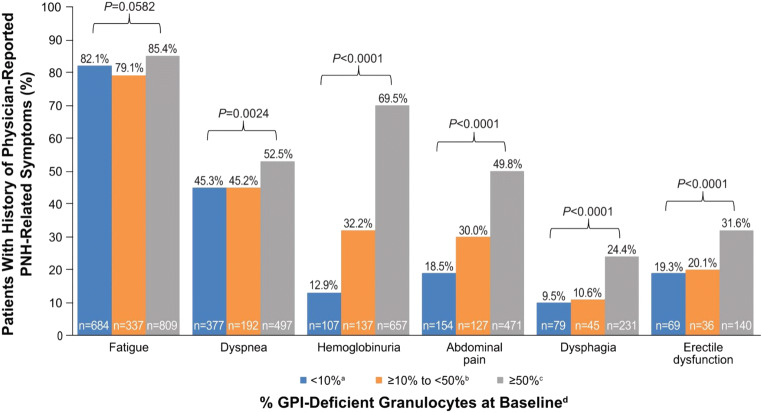
History of physician-reported PNH-related symptoms stratified by percentage of GPI-deficient granulocytes at baseline ^a^Fatigue, *n* = 833; dyspnea, *n* = 832; hemoglobinuria, *n* = 829; abdominal pain, *n* = 832; dysphagia, *n* = 830; erectile dysfunction, *n* = 357. ^b^Fatigue, *n* = 426; dyspnea, *n* = 425; hemoglobinuria, *n* = 425; abdominal pain, *n* = 424; dysphagia, *n* = 426; erectile dysfunction, *n* = 179. ^c^Fatigue, *n* = 947; dyspnea, *n* = 947; hemoglobinuria, *n* = 946; abdominal pain, *n* = 945; dysphagia, *n* = 946; erectile dysfunction, *n* = 443; ^d^Patients with available data. *GPI* glycosylphosphatidylinositol; *PNH* paroxysmal nocturnal hemoglobinuria

Substantial proportions of patients had a history of physician-reported PNH-related symptoms at baseline, including fatigue (80.9% [2684/3318]), dyspnea (45.3% [1501/3315]), hemoglobinuria (45.0% [1492/3313]), abdominal pain (35.2% [1167/3314]), dysphagia (16.5% [547/3311]), and erectile dysfunction (24.2% [344/1422 males]. Proportions of patients with PNH-related symptoms stratified by GPI-deficient granulocyte clone size at baseline are shown in Fig. 3. Although the proportion of patients with a history of each commonly reported PNH-related symptom was greatest in the subgroup with the largest GPI-deficient granulocyte clone size, a considerable proportion of patients with smaller clone sizes also experienced these symptoms.

In the overall population, patient-reported QoL assessments based on EORTC Global Health/QoL (*n* = 1888 patients with available data) and FACIT-Fatigue (*n* = 1894 patients with available data) scores indicated impaired QoL and fatigue, with median (Q1, Q3) scores of 58.3 (41.7, 75.0) and 34.0 (27.0, 40.0), respectively. Patient-reported QoL assessments did not appear to correlate with GPI-deficient granulocyte clone size. Median (Q1, Q3) FACIT-Fatigue scores (*n* = 1317 patients with available data) were 35.0 (28.0, 40.0) in patients with clone size < 10%, 37.0 (28.0, 41.0) in those with clone size ≥ 10% to < 50%, and 32.0 (23.0, 39.0) in those with clone size ≥ 50%; median (Q1, Q3) EORTC Global Health/QoL scores (*n* = 1310 patients with available data) were 58.3 (41.7, 75.0), 58.3 (50.0, 83.3), and 50.0 (33.3, 66.7), respectively.

## Discussion

The current study provides updated data from the International PNH Registry describing baseline characteristics and disease burden in a much larger cohort of patients (*n* = 4439) than previously reported (*n* = 1610) [6]. While the burden and/or natural history of PNH has been documented in smaller cohorts [1, 3, 6, 11, 15, 17, 21–24], the current analysis characterized the largest cohort of patients with PNH studied to date and examined trends in the relationship between disease burden and GPI-deficient granulocyte clone size.

The demographics and clinical history of the current study population were generally similar to the subset reported by Schrezenmeier et al. [6], although median age at PNH onset was slightly older and median duration of disease was slightly shorter in the current study. Median GPI-deficient granulocyte clone size was 31.8% in the present study compared with 68.1% in the earlier analysis of the Registry. In addition, our cohort had a higher proportion of patients with GPI-deficient granulocyte clone size < 10% compared with the previous study (37.1% versus 17.4%, respectively) and a smaller proportion of patients with clone size ≥ 50% (43.4% versus 51.7%, respectively) [6]. It is likely that more patients with small GPI-deficient granulocyte clone sizes have been enrolled in the Registry in recent years due to improvements in disease awareness, enhanced emphasis on screening (e.g., screening for GPI-deficient cells is now routine in patients with aplastic anemia) and early detection, and the increased availability and utilization of high-sensitivity flow cytometry for detection of PNH clones [4, 6, 25, 26].

Our findings confirmed that PNH is associated with a high burden of disease, as demonstrated by the proportion of patients who had HDA (51.6%), history of MAVEs (18.8%), TEs (13.3%), RBC transfusions (61.3%), and impaired renal function (eGFR < 90 mL/min/1.73 m^2^, 42.8%) at baseline. Although higher GPI-deficient granulocyte clone size correlated with a higher proportion of patients with HDA and history of MAVEs, a considerable proportion of patients in the cohorts with GPI-deficient granulocyte clone size ≥ 10% to < 50% and < 10% also had HDA and/or history of MAVEs, suggesting the need for further emphasis on disease symptoms in clinical decision-making and potential consideration of treatment in these populations.

The current analysis also examined the association of HDA with GPI-deficient granulocyte clone size, which was not previously assessed by Schrezenmeier et al. [6], and may be an important consideration in patient management. While more than half of the patients in this study had HDA [16], which was significantly and positively related to GPI-deficient granulocyte clone size, a considerable number of patients with small GPI-deficient granulocyte clone size also had HDA (9%). This finding is clinically relevant considering that previous research has demonstrated that presence of HDA is related to poorer outcomes and higher risk of TE [15, 16, 27].

The proportion of patients with a history of BMF was higher in this study compared with the previous study (62.6% versus 48.1%, respectively), potentially owing to the higher proportion of patients with GPI-deficient granulocyte clone size < 10% in the current study cohort as discussed above [6]. This observation is consistent with previous research showing that a majority of patients with concurrent PNH and BMF have GPI-deficient granulocyte clone sizes smaller than 10%, with less than 10% of patients with PNH/BMF having a clone size larger than 50% [28]. Of note, immunosuppressive therapy was reported in 59.0% of patients with GPI-deficient granulocyte clone size < 10%, and patients in this group had lower reticulocyte counts versus those with larger clone sizes, which is not unexpected given the high proportion of patients with a history of BMF in this group [13, 22, 29]. Interestingly, the cohort with the smallest GPI-deficient granulocyte clone sizes also had a higher proportion of patients with renal failure at baseline compared with the larger clone-size groups. This is probably due to the fact that the patients in the smallest GPI-deficient granulocyte clone-size group had the highest prevalence of concomitant BMF (89%) and immunosuppressive therapy use (59%) at baseline.

In the current analysis, 61.3% of patients had a history of RBC transfusions, compared with approximately 36% of patients in the previous Registry analysis [6]. This finding may be related to the higher proportion of patients with a history of BMF in the current study population, who often require supportive treatment with RBC transfusions [5]. However, we found no significant association between history of RBC transfusions and clone size, and more than half of the patients in all clone size groups had experienced RBC transfusions at baseline. It can be hypothesized that patients with larger GPI-deficient granulocyte clone size may receive RBC transfusions to treat anemia related to the hemolysis of PNH, whereas patients with a smaller clone size, who are more likely to have BMF, may receive transfusions to treat anemia associated with BMF [5].

A large proportion of patients had hemolysis (55.8%) and impaired renal function (42.8%) at baseline, and most laboratory values indicative of greater disease burden were positively correlated with GPI-deficient granulocyte clone size. Median LDH ratio and the proportion of patients with LDH ≥ 1.5 times ULN were significantly correlated to GPI-deficient granulocyte clone size, consistent with findings from Schrezenmeier et al. [6]. Interestingly, in our cohort, the proportions of patients with eGFR 30 to < 90 decreased with increasing GPI-deficient granulocyte clone size, whereas the proportions of patients with eGFR < 30 remained similar across different clone sizes. Although prior literature has demonstrated that renal dysfunction, including chronic kidney disease (CKD), is common among patients with PNH, additional analyses are needed to better understand the relationship between PNH and CKD and the impact of concurrent or previous nephrotoxic therapy (i.e., cyclosporine and treatment of BMF), and to identify potential trends related to GPI-deficient granulocyte clone size [30, 31].

A substantial proportion of patients in all GPI-deficient granulocyte clone size categories experienced physician-reported PNH-related symptoms such as fatigue, dyspnea, hemoglobinuria, abdominal pain, dysphagia, and erectile dysfunction. With the exception of fatigue, the proportions of patients with each symptom were significantly correlated with increasing GPI-deficient granulocyte clone size category, although a large proportion of patients with clone size < 50% experienced each symptom. This data pattern is similar to that observed by Schrezenmeier et al. [6], with the exception of dyspnea and abdominal pain, which were significantly correlated with GPI-deficient granulocyte clone size only in the current analysis. Although fatigue was the most commonly reported symptom, its presence was not associated with GPI-deficient granulocyte clone size in either analysis, suggesting that fatigue may not be a relevant indicator of disease severity based on clone size.

Finally, QoL assessment results from FACIT-Fatigue scores and EORTC Global Health/QoL (median [Q1, Q3], 34.0 [27.0, 40.0] and 58.3 [41.7, 75.0], respectively) indicated patients in our analysis experienced considerable disease-related fatigue and impairment in overall QoL relative to normative reference scores reported for the general adult population (mean [SD], 43.6 (9.4) and 75.7 (21.2), respectively) [19, 32]. Future analyses to better understand the relationships between these patient-reported outcomes and disease burden would provide further insight into the impact of PNH on patients’ health-related QoL.

Interpretation of our results may be mitigated by several limitations. The current analysis focused on patients with PNH who had not been treated with a complement inhibitor at the time of enrollment in the Registry, and we did not assess the potential impact of treatment on disease-related parameters. Because this analysis was based on an observational and noninterventional data set, not all patients had available data for every outcome assessed, and the current analysis did not account for changes in disease status that may have occurred prior to patients’ enrollment in the Registry. Thus, patients’ missing clone size data were not included in this analysis. Additional analyses are needed to investigate GPI-deficient granulocyte clone size and changes in clone size as potential prognostic markers for risk of clinical outcomes (e.g., MAVEs, including TEs). Given the heterogeneity in GPI-deficient granulocyte clone sizes observed in the current study, more robust prospective data are needed to confirm the best management approach for each individual patient with PNH. Also, comparisons across racial and/or ethnic subgroups were not conducted in the current study. The majority of patients in our analysis were white (78%) and from Europe (68%), with relatively small proportions of Asians (16%) and blacks (3%) represented in the study population. In a previous report of PNH Registry data from patients with PNH in South Korea, median granulocyte clone size was higher (48.8% versus 31.8%), as was median LDH ratio (4.1 versus 1.9) than the overall patient population in the current study [15]. Therefore, our observed results should not be extrapolated to race- or ethnicity-based subgroups.

In summary, consistent with previous analyses of patients in the PNH Registry, higher disease burden was generally associated with larger GPI-deficient granulocyte clone size in our study. However, substantial disease burden was still found in patients with GPI-deficient granulocyte clone size < 10%. The presence of hemolysis in patients of all GPI-deficient granulocyte clone sizes is of particular clinical relevance because hemolysis is associated with significantly higher risk of TEs, the leading cause of mortality in PNH [15, 17, 27]. In this study, a noteworthy proportion of patients with GPI-deficient granulocyte clone size < 10% experienced HDA, MAVEs, RBC transfusions, impaired renal function, hemolysis, and PNH-related symptoms, which might also be associated with the underlying BMF.

## Conclusions

This International PNH Registry analysis is the largest PNH study to date to document the clinical characteristics, burden of disease, and potential trends in manifestations of PNH related to GPI-deficient granulocyte clone size. In general, patients with larger GPI-deficient granulocyte clone sizes had higher disease burden; however, many patients with smaller clone sizes had experienced clinically important medical events and associated symptoms. This study confirms the findings of a previous Registry analysis [6] and adds to our knowledge regarding the prevalence of HDA in Registry patients as well as the relationship between HDA and GPI-deficient granulocyte clone size in patients with PNH. The improved characterization of this rare, life-threatening disease and of relationships among clinical measures of interest in patients with PNH may better inform patient management.
